# Residual CatLet score and its derived indexes in predicting outcomes in patients with chronic coronary syndrome after percutaneous coronary intervention

**DOI:** 10.3389/fcvm.2026.1780523

**Published:** 2026-03-25

**Authors:** Juan Wang, Mingchao Zhang, Fan Zhang, Jiehua Li, Deguo Wang

**Affiliations:** 1 Department of Cardiology, The First Affiliated Hospital of Anhui Medical University, Hefei, China; 2 The Second Affiliated Hospital of Wannan Medical College, Wuhu, China; 3 Department of Cardiology, The Second Affiliated Hospital of Wannan Medical College, Wuhu, China; 4 Department of Cardiac Rehabilitation, The Second Affiliated Hospital of Wannan Medical College, Wuhu, China; 5 The First Affiliated Hospital of Wannan Medical College, Wuhu, China

**Keywords:** CatLet revascularization index, chronic coronary syndrome, prognosis, residual CatLet score, risk

## Abstract

**Background:**

Coronary Artery Tree description and Lesion EvaluaTion (CatLet) angiographic scoring (CS) system, reflecting coronary artery variability, is a newly developed scoring tool for assessing the degree of coronary artery stenosis. And the residual CatLet score (rCS) has reflected the burden of residual lesions. This study was conducted to explore the utility of the rCS and its derived indexes-Catlet revascularization index (CRI) among patient with chronic coronary syndrome (CCS) after percutaneous coronary intervention (PCI).

**Methods:**

A study cohort was established comprising patients who received PCI in the Second Affiliated Hospital of Wannan Medical College from May 2019 to July 2020. Patients were divided into tertiles according to the CRI and rCS values. The primary endpoint was major adverse cardiac events (MACEs), including myocardial infarction, cardiac death, heart failure and all-cause mortality, secondary outcome measures were recurrent angina.

**Results:**

A total of 262 patients were enrolled in this analysis, with mean CRI and rCS scores of 58.08 ± 27.88 and 17.33 ± 15.81, respectively. Kaplan–Meier analysis revealed that higher levels of residual coronary lesions, as measured by rCS and its derivatives, correlated with a higher incidence of adverse cardiovascular events. Multivariable Cox regression analysis demonstrated that CRI [hazard ratio (HR): 0.98; 95% confidence interval (CI): 0.98–0.99; *p* < 0.001] and rCS (HR: 1.04; 95% CI: 1.02–1.05; *p* < 0.001) were significant independent predictors of MACEs during long-term follow-up; CRI (HR 0.98, 95% CI 0.98–0.99; *p* < 0.001) and rCS (HR 1.05, 95% CI 1.03–1.07; *p* < 0.001) emerged as significant predictors for the risk of recurrent angina. rCS demonstrated superior discriminative ability for MACEs compared to CRI at 6.2 years, with area under the curve values of 0.73 and 0.67, respectively (*p* < 0.001).

**Conclusion:**

This real-world analysis of CCS patients undergoing PCI demonstrated that the rCatlet score offered superior predictive performance for long-term MACEs compared to the CRI.

## 1 Introduction

In hemodynamically stable patients, achieving complete revascularization (CR) after percutaneous coronary intervention (PCI) is linked to better prognosis for those with either chronic coronary syndromes (CCS) or acute coronary syndromes (1–3). However, incomplete revascularization (IR) is a common consequence of PCI, especially those with CCS, often due to complex coronary anatomy or serious comorbid conditions (4–6). The reported prognostic impact of incomplete revascularization (IR) after PCI has been heterogeneous across studies (7–9), in part because of the absence of a standardized definition for IR.

The residual SYNTAX Score and its derived indexes SYNTAX revascularization index (SRI) enables more precise evaluation of post-PCI residual stenosis and lesion complexity, thereby providing deeper insights into how incomplete revascularization affects patient prognosis (10–12). However, by restricting coronary dominance to a binary classification, the SYNTAX score fails to capture the full spectrum of anatomical variability and falls short to semiquantify the extent of coronary blood supply (13). The recently introduced CatLet score system incorporates the capability to account for anatomical variability in the coronary tree, evaluate the severity of arterial lesions, and semiquantify the extent of compromised blood supply, thus providing a comprehensive assessment of coronary artery disease including AMI or CCS (14–18).

Previous research has demonstrated that integrating clinical variables such as age, left ventricular ejection fraction (LVEF), and serum creatinine with resudial CatLet score has been shown to enhance its predictive value in patients with AMI (19). To date, few studies have explored the utility of rCatlet score and its derived indexes-CatLet revascularization index (CRI) among the unselective real-world CCS patients undergoing contemporary PCI treatment. The present study was conducted to assess and compare the prognostic capacity of two indexes in patients undergoing PCI in daily practice.

## 2 Methods

### 2.1 Study patients

This was a single-center, retrospective cohort study, a cohort of 300 patients with a diagnosis of CCS was initially enrolled in this study. All patients had received PCI at the Department of Cardiology, the Second Affiliated Hospital of Wannan Medical College, from May 2019 to July 2020. The CatLet score (CS) and the rCS from all coronary angiograms, using standard quantitative coronary analysis methodology, were assessed by an independent angiographic system blinded to clinical outcomes. The CRI was calculated with the following formula:CRI = (1-rCS/baseline CS) × 100. Thirty-eight patients were excluded based on the following criteria: (1) stent thrombosis within 1 month after PCI; (2) insufficient clinical or angiographic data; (3) loss to follow-up; (4) normal CAG results; (5) patients with coronary artery bypass graft surgery.

This study was performed in accordance with the Declaration of Helsinki. The Ethics Committee of the Second Affiliated Hospital of Wannan Medical College granted a waiver of informed consent due to the retrospective nature of the research.

### 2.2 Procedures and medications

Treatment strategies, including PCI approach and stent selection, were determined by the interventional cardiologist. Preprocedural antiplatelet therapy was standardized: patients not on long-term aspirin and a P2Y12 inhibitor received a loading dose (aspirin 300 mg and clopidogrel 300 mg or ticagrelor 180 mg) at least 24 h prior to elective PCI. An intraprocedural bolus of unfractionated heparin (100 U/kg) was administered to all patients, with glycoprotein IIb/IIIa inhibitor use at the operator's discretion. Post-procedure, all patients were prescribed lifelong aspirin (100 mg daily) plus either clopidogrel (75 mg daily) or ticagrelor (90 mg twice daily) for a minimum of 1 year.

### 2.3 Sample size estimation

Based on prior studies, each 1-unit increase in the CatLet score was associated with a hazard ratio of 1.05, with a standard deviation of 12.0 (15). Previous reports also indicated a failure probability of approximately 15% at 1.5 years (20). Assuming a power of 0.9 and a two-sided alpha of 0.05, a minimum sample size of 205 is required to ensure robust conclusions.

### 2.4 The CatLet score and the lesion evaluation

**Figure 1:**
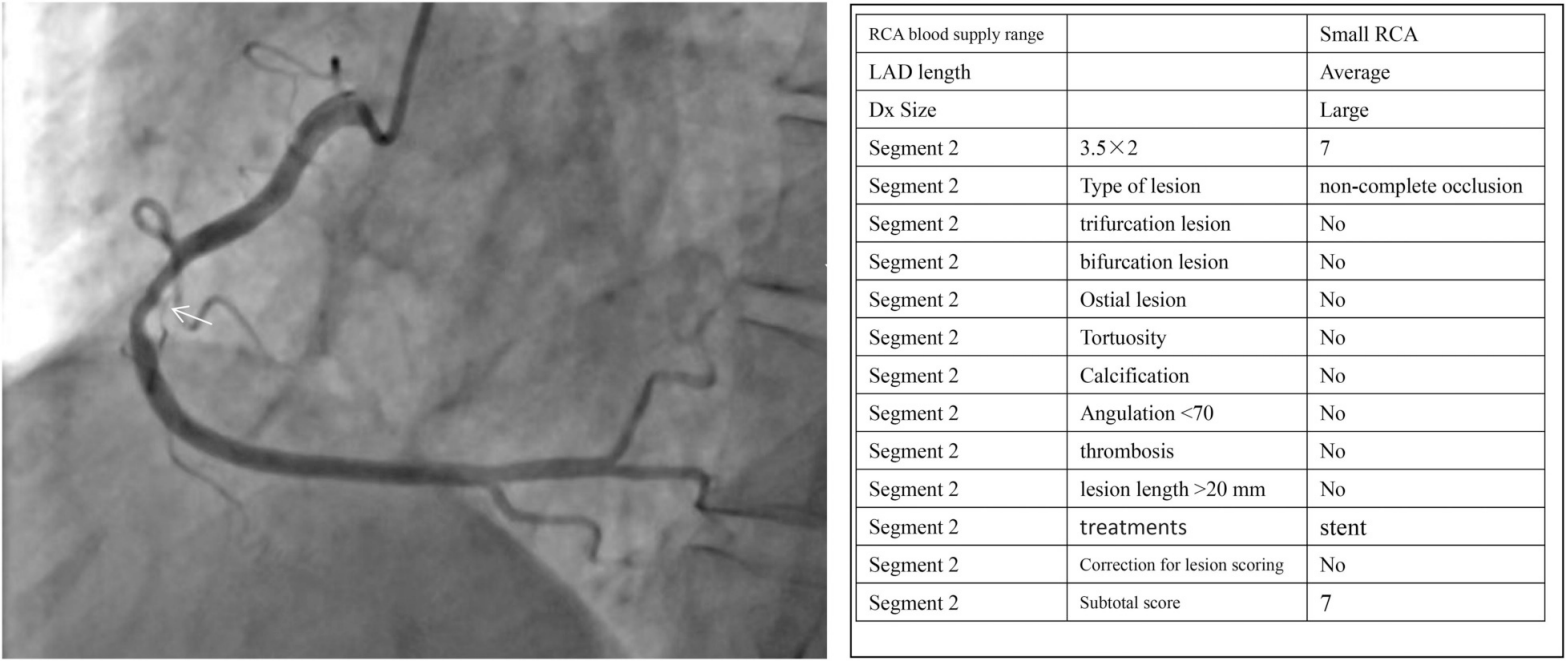
An example of the CatLet score (the non-occlusive lesion on mid RCA).

The CatLet angiographic scoring system, which has been extensively detailed in a previous publication (14), is accessible online along with instructional tutorials and calculation tools at http://www.catletscore.com. This scoring system integrates a 17-segment myocardial model, the principle of competitive blood supply, and the law of flow conservation. A key characteristic of the system is its incorporation of coronary anatomical variability and the extent of myocardial blood supply affected by diseased vessels. Based on its scoring rules, coronary circulation patterns are classified into 54 possible categories, derived from 6 types of right coronary artery anatomy, 3 types of left anterior descending branch anatomy, and 3 types of diagonal branch anatomy. Each lesion is scored by multiplying a weighting factor by a stenosis coefficient-2.0 for 50%–99% diameter stenosis and 5.0 for total occlusion. The total coronary lesion score represents the sum of all individual lesion scores. Only lesions in vessels ≥1.5 mm in diameter with ≥50% stenosis are scored, with adjustments made as necessary. Adverse lesion characteristics are documented objectively but are not incorporated into the score. Non-occlusive lesions do not receive an occlusion status score, whereas totally occluded lesions are evaluated using three scoring scenarios: (1) occlusive score; (2) guidewire passage/non-occlusion score after balloon dilation; and (3) score after stenosis removal (16). A representative example of the CatLet score calculation is provided in Figure 1. The residual CatLet score was calculated after the completion of all intended revascularization. For patients undergoing a single PCI session, the score was determined immediately after that procedure. For those with a planned staged PCI for non-culprit lesions, the score was calculated after the final procedure. The inter- and intra-observer reproducibility of the CatLet score has been formally validated in a dedicated study, demonstrating substantial to excellent agreement (weighted kappa values) among interventional cardiologists (21). In this study, two interventional cardiologists independently scored all coronary angiograms. Discrepancies were resolved through discussion with a third analyst. All observers were blinded to the clinical data and outcomes of the patients.

### 2.5 Disease definitions and endpoints

Patient diagnoses and endpoint definitions were established as follows. Chronic coronary syndrome (CCS) was diagnosed in patients with atherosclerotic heart disease or corresponding risk factors, primarily based on a typical angina history (22). All undetermined deaths were adjudicated as cardiac in origin unless a non-cardiac cause was definitively established by clinical or autopsy evidence (23). AMI was defined according to the third universal definition of myocardial infarction (24). Recurrent angina was defined as the recurrence of typical anginal chest pain after discharge, resulting in the need for intensification of antianginal medical therapy or leading to repeat coronary angiography with or without subsequent revascularization. The diagnosis required confirmation by the treating physician and was supported, where available, by new ischemic electrocardiographic changes or a positive stress test. Heart failure was defined according to the universal definition of heart failure (25). The primary endpoint, major adverse cardiovascular events (MACEs), was defined as a composite of all-cause death, myocardial infarction, cardiac death, any revascularization, or heart failure occurring during the follow-up, secondary outcome measures were recurrent angina. Procedural success was defined as successful stent implantation with a final residual stenosis of <50% and Thrombolysis in Myocardial Infarction (TIMI) flow grade 3. All endpoint events were centrally adjudicated by two independent cardiologists, with any disagreements settled through consensus.

### 2.6 Follow-up

Patient follow-up was conducted via structured telephone interviews with surviving patients or their immediate family members to ascertain the occurrence of major adverse cardiac events (MACEs). All patients were followed until the occurrence of a MACE or the study's conclusion (May 2025), whichever came first. For patients who experienced adverse events, relevant medical records, discharge summaries, and imaging data were retrieved and reviewed. Information regarding death was obtained from hospital records or confirmation by next of kin.

### 2.7 Statistical analysis

Continuous variables are expressed as medians (interquartile range, IQR). Categorical variables are expressed in terms of frequency (percentage). Statistical analyses were performed to assess trends in event incidence at all levels using Stata software (StataCorp, College Station, TX, USA). Missing data were addressed using multiple imputation. Survival curves were depicted using the Kaplan–Meier method and compared with the log-rank test. To identify independent predictors of clinical outcomes, we performed Cox regression survival analyses. The selection of covariates for adjustment was based on clinical relevance and some significant association (*p* < 0.05) with the outcome in univariate analysis. The final adjusted models included the following variables: age, Creatinine, left ventricular ejection fraction, neutrophil to lymphocyte ratio, adverse lesion characteristics as calcification, Tortuosity, Trifuration and stent length. Results are presented as adjusted hazard ratios (aHR) with 95% confidence intervals (CI). Model performance was assessed by its discrimination and calibration, with discrimination compared between scores using the receiver operating characteristic (ROC) curves. The interaction between the continuous rCatLet score and other risk factors was evaluated using a *z*-test (26). All analyses were conducted using Stata version 17. Statistical tests were two-sided, and a *p* < 0.05 was considered statistically significant.

## 3 Results

### 3.1 Baseline characteristics

**Figure 2:**
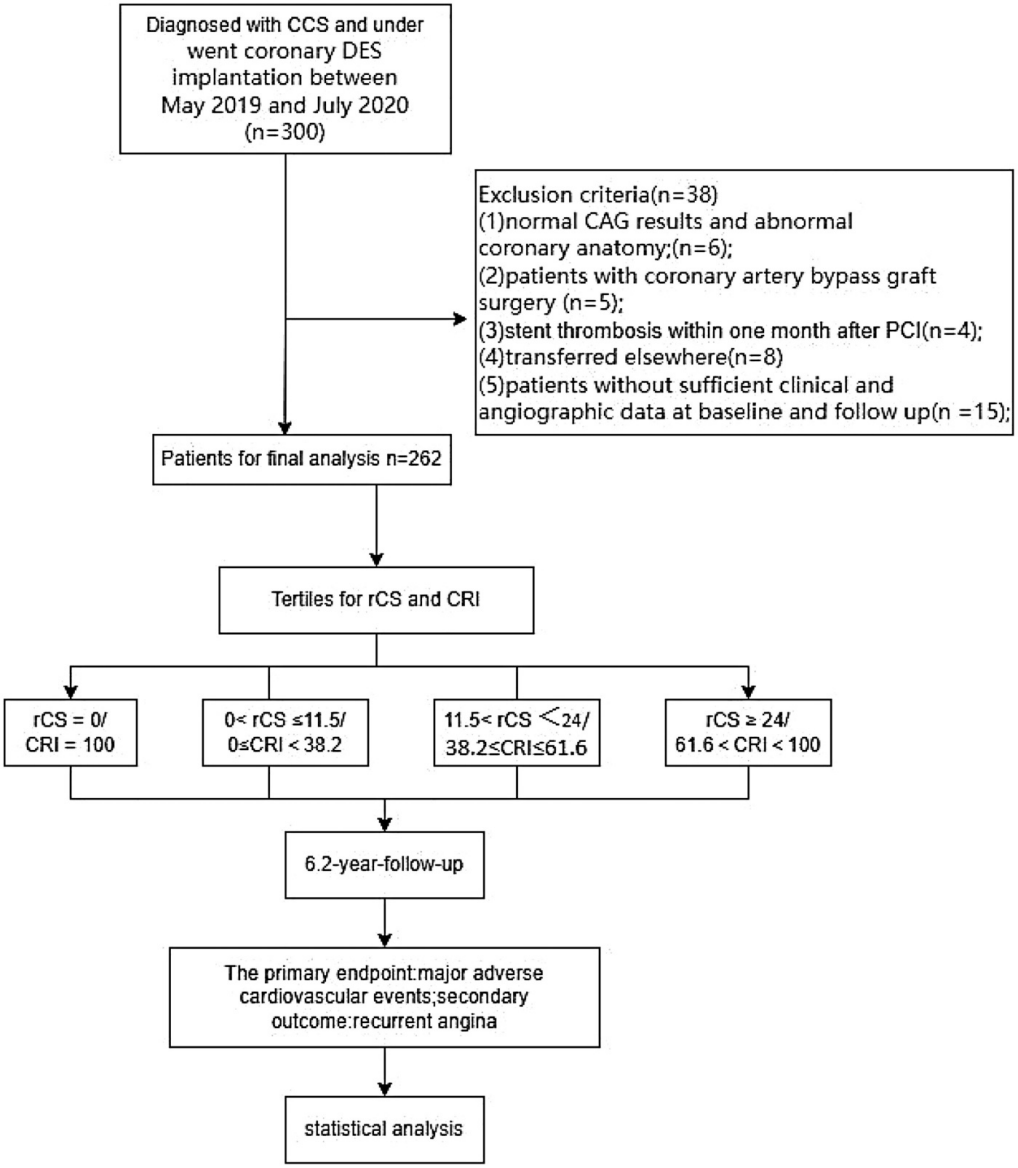
Patient study flow diagram.

A total of 262 CCS patients treated with PCI were enrolled as shown in Figure 2. Among them, 29 patients had chronic total occlusion lesions on angiography. A minority of patients (16.8%, 44/262) achieved complete revascularization (CR; rCS = 0 and CRI = 100). The majority who underwent incomplete revascularization (IR) were further stratified into three groups according to rCS and CRI tertiles [Tables 1, 2 and Supplementary Tables S1, S2]. The median age was 67 (IQR: 59–75), and 71.0% (186/262) were male. The rCatLet score ranged from 0 to 75.5, with a mean ± SD of 17.3 ± 15.8 and a median of 14 (IQR: 5–24). Patients in higher rCatLet score tertiles had lower left ventricular ejection fraction, more likely to smoke, more adverse lesion characteristics including the number of lesion, stent diameter etc., higher baseline CS and higher neutrophil-to-lymphocyte ratios.

**Table 1:** Baseline characteristics of all patients after PCI according to rCS.

Variables	rCS = 0	0 < rCS ≤ 11.5	11.5 < rCS < 24	rCS ≥ 24	*p*-value
No. of cases	44	69	74	75	
Age (years)	67.14 (11.28)	64.30 (9.85)	66.93 (10.51)	67.85 (10.86)	0.22
Male	30 (68%)	52 (75%)	51 (69%)	53 (71%)	0.81
BMI	23.30 (3.52)	24.08 (3.00)	24.21 (3.36)	23.63 (4.03)	0.48
Medical history					
Diabetes	11 (25%)	19 (28%)	22 (30%)	29 (39%)	0.35
Hypertension	30 (68%)	44 (64%)	54 (73%)	55 (73%)	0.56
CKD	3 (7%)	1 (1%)	6 (8%)	7 (9%)	0.24
Smoking					0.034
Current	11 (25%)	34 (49%)	27 (36%)	36 (48%)	
Never	21 (48%)	30 (43%)	38 (51%)	31 (41%)	
Past	12 (27%)	5 (7%)	9 (12%)	8 (11%)	
Alcohol consumption					0.42
Current	5 (11%)	18 (26%)	13 (18%)	16 (21%)	
Never	35 (80%)	48 (70%)	57 (77%)	57 (76%)	
Past	4 (9%)	3 (4%)	4 (5%)	2 (3%)	
Laboratory results					
LVEF	61.0 (8.00)	60.0 (9.00)	59.0 (9.00)	54.0 (11.0)	<0.001
Creatinine, umol/L	92.41 (24.03)	95.84 (37.88)	105.42 (48.66)	118.97 (98.03)	0.074
TG, mmol/L	1.72 (1.11)	1.81 (1.17)	1.78 (0.83)	1.55 (0.88)	0.39
TC, mmol/L	4.35 (0.90)	4.73 (1.20)	4.40 (1.01)	4.42 (1.21)	0.20
HDL-C, mmol/L	1.17 (0.49)	1.09 (0.27)	1.08 (0.33)	1.03 (0.27)	0.16
LDL-C, mmol/L	2.48 (0.79)	2.78 (0.92)	2.57 (0.87)	2.65 (1.00)	0.35
FBG, mmol/L	6.22 (1.96)	6.41 (2.38)	5.99 (1.57)	7.15 (3.37)	0.032
NLR	3.06 (1.76)	3.08 (2.41)	3.43 (2.38)	4.77 (5.02)	0.007

Data are expressed as mean ± standard deviation or *n* (%). CKD, chronic kidney disease; HDL-C, high density lipoprotein cholesterol; LDL-C, low-density lipoprotein cholesterol; LVEF, left ventricular ejection fraction; FBG, fasting blood glucose; rCS, residual CatLet score; TC, total cholesterol; TG, triglyceride; UA, uric acid; NLR, neutrophil to lymphocyte ratio.

**Table 2:** Baseline angiographic characteristics of all patients after PCI according to rCS.

Variables	rCS = 0	0 < rCS ≤ 11.5	11.5 < rCS < 24	rCS ≥ 24	*p*-value
No. of cases	44	69	74	75	
Diagonal size					0.90
Small	4 (9%)	5 (7%)	6 (8%)	9 (12%)	
Inter	14 (32%)	19 (28%)	21 (28%)	17 (23%)	
Large	26 (59%)	45 (65%)	47 (64%)	49 (65%)	
LAD length					0.42
Short	7 (16%)	10 (14%)	14 (19%)	19 (25%)	
Average	18 (41%)	22 (32%)	22 (30%)	30 (40%)	
Long	19 (43%)	37 (54%)	38 (51%)	26 (35%)	
RCA dominance					0.77
PDA zero	2 (5%)	3 (4%)	2 (3%)	1 (1%)	
PDA only	2 (5%)	3 (4%)	3 (4%)	5 (7%)	
SmallRCA	11 (25%)	20 (29%)	18 (24%)	15 (20%)	
AverageRCA	20 (45%)	35 (51%)	41 (55%)	36 (48%)	
LargeRCA	9 (20%)	8 (12%)	10 (14%)	17 (23%)	
SuperRCA	0 (0%)	0 (0%)	0 (0%)	1 (1%)	
Coronary artery treated					
LM	1 (2%)	0 (0%)	3 (4%)	5 (7%)	0.17
LAD	31 (70%)	51 (74%)	50 (68%)	41 (55%)	0.082
LCX	6 (14%)	11 (16%)	18 (24%)	17 (23%)	0.39
RCA	6 (14%)	16 (23%)	26 (35%)	25 (33%)	0.041
Culprit vessels					
LAD	30 (68%)	47 (68%)	40 (54%)	37 (49%)	0.057
LCX	6 (14%)	7 (10%)	10 (14%)	11 (15%)	0.87
RCA	6 (14%)	15 (22%)	22 (30%)	23 (31%)	0.14
Bifurcation	20 (45%)	48 (70%)	42 (57%)	43 (57%)	0.082
Trifuration	1 (2%)	1 (1%)	5 (7%)	9 (12%)	0.040
Tortuosity	11 (25%)	17 (25%)	30 (41%)	44 (59%)	<0.001
Chronic total occlusion	1 (2%)	3 (4%)	7 (9%)	18 (24%)	<0.001
Calcification	2 (5%)	1 (1%)	8 (11%)	10 (13%)	0.038
Ostial lesion	4 (9%)	8 (12%)	12 (16%)	12 (16%)	0.62
Post-dilation	25 (57%)	47 (68%)	51 (69%)	52 (69%)	0.49
Angulation <70	18 (41%)	48 (70%)	37 (50%)	36 (48%)	0.011
lesion length >20 mm	28 (64%)	54 (78%)	63 (85%)	63 (84%)	0.027
total stent number	1.18 (0.58)	1.32 (0.53)	1.53 (0.83)	1.53 (0.74)	0.017
total lesion number	1.14 (2.07)	2.07 (0.73)	2.77 (0.63)	3.07 (0.55)	<0.001
Baseline Catlet score	14.68 (8.53)	24.80 (13.57)	38.74 (14.28)	56.28 (17.33)	<0.001
Minimal stent diameter, mm	3.16 (0.45)	3.04 (0.36)	2.98 (0.44)	2.84 (0.43)	<0.001

Data are expressed as mean ± SD or *n* (%). LAD, left anterior descending; LCX, left circumflex artery; LM, left main; RCA, right coronary artery; rCS, residual CatLet score; PDA, posterior descending artery.

### 3.2 Clinical outcomes

**Figure 3:**
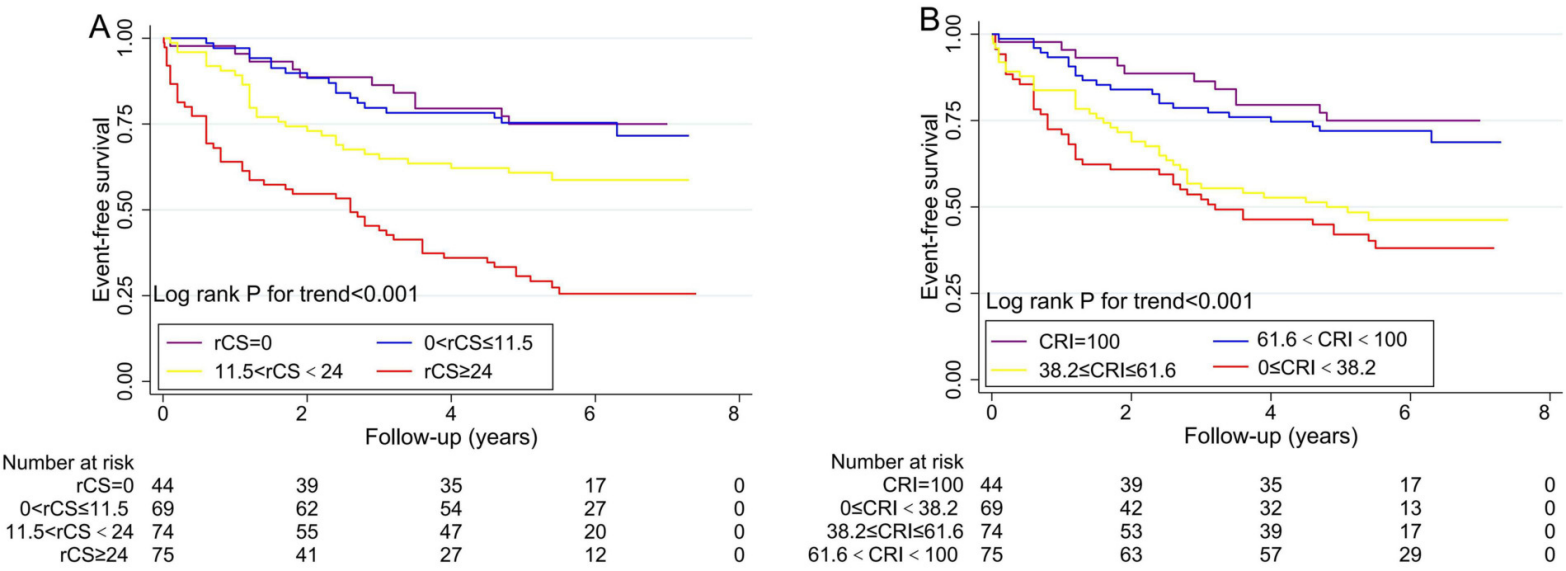
Kaplan–Meier analysis illustrates event-free survival for the incidence of the primary outcome in the subgroups stratified by **(A)** residual CatLet score (rCS) and **(B)** CatLet revascularization index (CRI) during a 6.2-year follow-up.

During a median follow-up of 42 months, the primary outcome occurred in 114 patients (43.5%) of the overall cohort, while recurrent angina was observed in 68 patients (26.0%) (Table 3). The Kaplan–Meier curves in Figure 3 depict the incidence of the primary outcome across subgroups stratified by rCS and CRI tertiles.

**Table 3:** Clinical outcome of the overall study population and of the subgroups stratified according to the CRI and rCS tertiles.

Adverse events	Overall (262)	CRI = 100 (*n* = 44)	0 ≤ CRI < 38.2 (*n* = 74)	38.2 ≤ CRI ≤ 61.6 (*n* = 72)	61.6 < CRI < 100 (*n* = 72)	p* value	rCS = 0 (*n* = 44)	0 < rCS ≤ 11.5 (*n* = 69)	11.5 < rCS < 24 (*n* = 74)	rCS ≥ 24 (*n* = 75)	p* value
MACEs	114 (43.5)	11 (25.0)	43 (58.1)	38 (52.8)	22 (30.6)	<0.001	11 (25.0)	18 (26.1)	30 (40.5)	55 (73.3)	<0.001
Cardiac death	11 (4.2)	0 (0.0)	5 (6.8)	5 (6.9)	1 (1.4)	0.117	0 (0.0)	0 (0.0)	3 (4.1)	8 (10.7)	0.005
All-cause death	4 (1.5)	2 (4.6)	0 (0.0)	1 (1.4)	1 (1.4)	0.280	2 (4.6)	1 (1.5)	0 (0.0)	1 (1.3)	0.28
Heart failure	25 (9.5)	4 (9.1)	6 (8.1)	9 (12.5)	6 (8.3)	0.792	4 (9.1)	6 (8.7)	4 (5.4)	11 (14.7)	0.282
Recurrent angina	68 (26.0)	5 (11.4)	28 (37.8)	21 (29.2)	14 (19.4)	0.006	5 (11.4)	10 (14.5)	23 (31.1)	30 (40.0)	<0.001

Categorical variables are expressed as *N* (%). MACEs, major adverse cardiac events; CRI, Catlet revascularization index; rCS, residual Catlet score. P* indicates comparison between different residual CatLet score and CRI categories.

**Figure 4:**
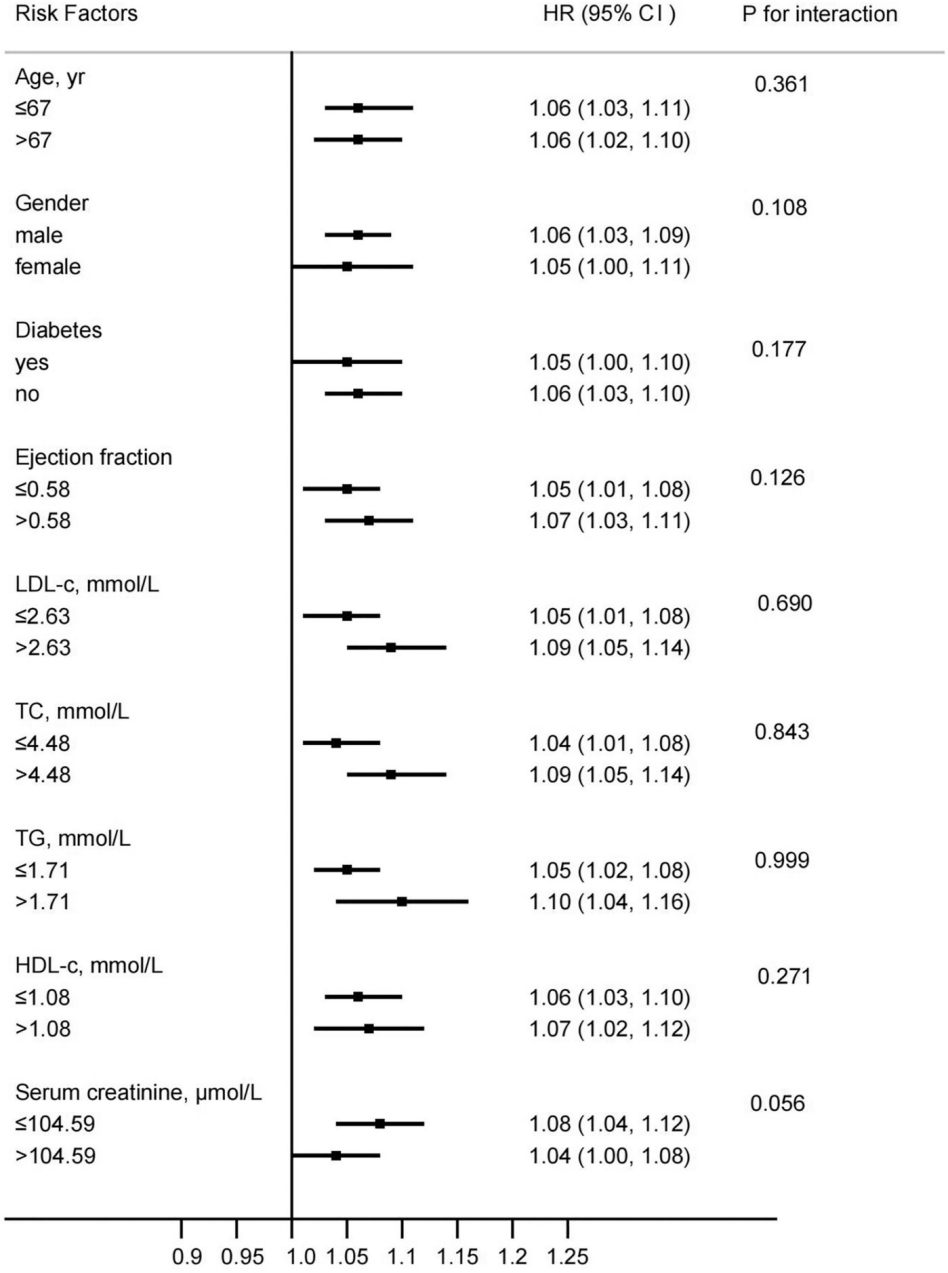
Hazard ratios for MACEs per 1 unit higher rCatLet score stratified by risk factors, categorically or medially, adjusted for creatinine, ejection fraction, neutrophil-to-lymphocyte ratio, lenth of stents, calcification, tortuosity, trifurcation, and minimal stent diameter.

Each unit increase in the rCS was independently associated with a higher risk of both MACE (adjusted HR 1.04, 95% CI 1.02–1.05; *p* < 0.001) and recurrent angina (adjusted HR 1.05, 95% CI 1.03–1.07; *p* < 0.001). Conversely, each unit increase in the CRI was associated with a lower risk for both endpoints (adjusted HR 0.98, 95% CI 0.98–0.99 for MACE; adjusted HR 0.98, 95% CI 0.98–0.99 for recurrent angina; both *p* < 0.001) (Table 4). Subgroup analysis had demonstrated that the rCatLet score was a consistent hazard risk for MACE, which deserved further study as shown in Figure 4.

**Table 4:** Unadjusted and adjusted Cox regression analysis for the primary and secondary outcome measures.

Adverse events	Score	HR	CI	*p* values on trend
Unadjusted				
MACEs	CRI	0.98	0.98–0.99	<0.001
	rCS	1.04	1.03–1.05	<0.001
Cardiac death	CRI	0.96	0.94–0.99	0.002
	rCS	1.06	1.04–1.09	<0.001
All-cause death	CRI	1.03	0.99–1.08	0.126
	rCS	0.95	0.85–1.06	0.291
Heart failure	CRI	0.99	0.98–1.01	0.335
	rCS	1.03	1.01–1.05	0.009
Recurrent angina	CRI	0.98	0.98–0.99	<0.001
	rCS	1.04	1.03–1.05	<0.001
Adjusted				
MACEs	CRI	0.98	0.98–0.99	<0.001
	rCS	1.04	1.02–1.05	<0.001
Cardiac death	CRI	0.98	0.95–1.01	0.136
	rCS	1.00	0.96–1.04	0.936
All-cause death	CRI	1.04	0.98–1.09	0.181
	rCS	0.95	0.84–1.06	0.361
Heart failure	CRI	1.00	0.99–1.02	0.548
	rCS	1.01	0.98–1.04	0.61
Recurrent angina	CRI	0.98	0.98–0.99	<0.001
	rCS	1.05	1.03–1.06	<0.001

CI, confidence interval; HR, hazard ratio; MACEs, major adverse cardiac events; CRI, Catlet revascularization index; rCS, residual Catlet score.

### 3.3 Predictive capability of residual CatLet score and Catlet revascularization index

**Figure 5:**
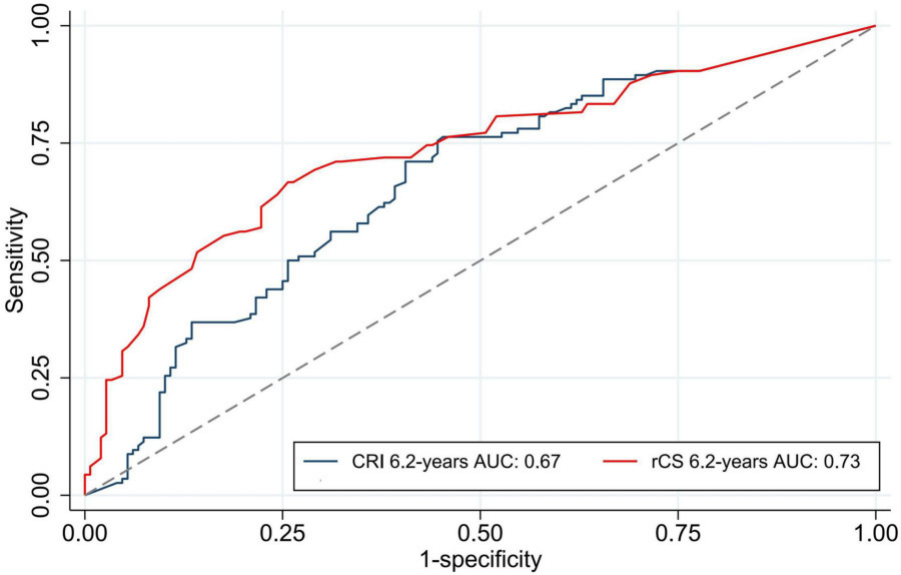
ROC analysis with AUC values of the rCS and CRI for MACEs at 6.2 years follow-up. AUC, area under the curve; ROC, receiver operating characteristic.

**Figure 6:**
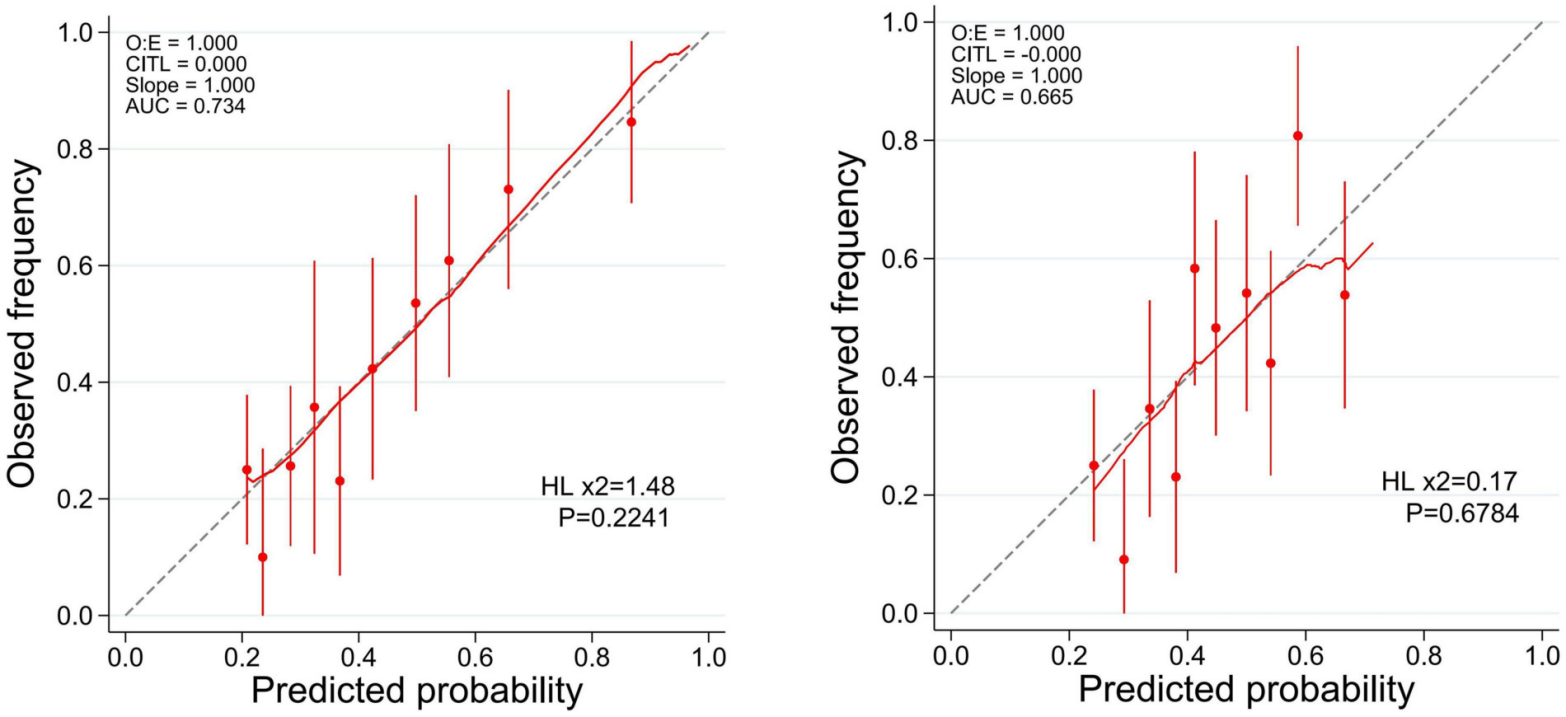
Calibration plots of univariate models for predicting major adverse cardiac events (MACEs). A red LOWESS smoothing curve is superimposed on each plot. Ideal calibration corresponds to an intercept of 0 and slope of 1. HL, Hosmer-Lemeshow test.

ROC analysis at 6.2 years revealed that the rCS exhibited superior predictive performance for MACE compared to the CRI, with AUCs of 0.73 vs. 0.67, respectively (*p* < 0.001; Figure 5). Furthermore, calibration plots indicated that both the rCatLet score and the CRI model were well-calibrated (Figure 6).

## 4 Discussion

This real-world study investigated the long-term prognostic value of two coronary anatomical indices—residual CatLet score (rCS) and CatLet revascularization index (CRI)—in patients with chronic coronary syndrome (CCS) undergoing percutaneous coronary intervention (PCI). The principal findings of this study are as following: (1) Both the rCS and CRI were independent predictors of MACE and recurrent angina. (2) The rCS demonstrated superior long-term prognostic performance for adverse events compared to the CRI.

The rate of CR in this unselected real-world PCI cohort was 16.8%, which is lower than reported in some previous studies (27, 28). This finding is understandable given the differences in study populations. While prior research often focused on patients with acute coronary syndromes and less complex coronary lesions, our real-world cohort included more complex cases, including chronic total occlusions (11.07%), a subset in which achieving complete revascularization is frequently challenging. Our analysis revealed that patients with a greater burden of residual coronary disease, quantified by higher rCS and its derived indexes, presented with a higher-risk clinical profile. This profile included a greater likelihood of smoking, reduced renal function, lower LVEF, elevated neutrophil-to-lymphocyte ratios, more adverse lesion characteristics including the number of lesion, stent diameter etc., and a higher baseline CatLet score. This clustering of risk factors reflects the well-documented clinical phenomenon whereby higher-risk patients are more likely to undergo incomplete revascularization (IR), just as prior studies indicated (19).

Following the widespread adoption of culprit-lesion PCI, attention has increasingly turned to the prognostic implications of the residual disease burden. Prior studies have established the predictive value of the residual Catlet score (rCS) in patients with AMI undergoing PCI, identifying a cut-off of rCS > 11 as being strongly associated with increased mortality and MACE events (19). In this context, the residual SYNTAX score, a metric detailed by Généreux et al. (29), emerged as a powerful predictor of coronary events and all-cause mortality in PCI patients (30). Furthermore, novel indexes derived from the rSS have subsequently been developed to quantify revascularization completeness after PCI. Among these, the SYNTAX Revascularization Index (SRI), which reflects the proportion of treated baseline lesions, has been established as an independent predictor of post-PCI mortality and MACE events (31, 32), a SRI ≥ 65% might be a reasonable threshold of incomplete revascularization for patients who underwent PCI treatment (31). Given that the CatLet score semiquantifies the severity and complexity of coronary artery disease (14), and the rCatLet score has reflected the burden of residual lesions. It is noteworthy that a prior study conducted in a cohort of patients with acute myocardial infarction (AMI) after PCI, has already performed a head-to-head comparison between the baseline CatLet score and the SYNTAX score, which demonstrated that the CatLet score exhibited superior calibration and risk stratification ability compared to the SYNTAX score for predicting clinical outcomes (15, 33). Therefore, we believe the residual Catlet score exerts the same effect. Consistent with previous reports in AMI populations (19), our results corroborate the prognostic utility of the rCatLet score. In our cohort, each unit increase in the score was associated with a significantly higher risk of MACE, corresponding to a hazard ratio of 1.04 (*p* < 0.001). Our analysis provides compelling evidence for the prognostic utility of the rCS and its derived indexes in a real-world PCI cohort, as all demonstrated significant ability to stratify the risk of 6.2-year adverse cardiovascular events. A prior study has validated the reproducibility of CatLet score in an AMI population (21), supporting its applications in the current study although we have to acknowledge that an independent reproducibility study on the CatLet score is an unmet need in this CCS population. These tools thereby offer clinicians practical means to objectively quantify the completeness of revascularization and to estimate subsequent patient prognosis.

## 5 Limitation

This study has several limitations. First, its observational, single-center design with a limited sample size may affect the generalizability of the findings and reduce statistical power. Second, although we adjusted for known confounders, unmeasured residual confounding cannot be excluded due to the non-randomized design; thus, the results should be considered hypothesis-generating. Fourth, In addition to conventional pharmacotherapy, nutraceuticals represent a potential modulator of cardiovascular risk in patients with chronic coronary syndrome (CCS), as evidenced by Scicchitano et al. (34). These compounds exert beneficial effects on lipid metabolism, endothelial function, and systemic inflammation—key pathophysiological processes that may influence the prognostic stratification capacity of the CatLet score. It should be noted, however, that the present study lacks systematic documentation of nutraceutical consumption or dietary habits. This constitutes a known methodological constraint, as such non-pharmacological interventions could potentially attenuate residual risk factors not accounted for in our predictive model. Furthermore, future studies could assess the reproducibility of the residual CatLet score within a CCS-specific cohort to confirm its consistency in this particular clinical context, although its foundational methodology has already demonstrated excellent agreement in prior evaluations. Besides, it is important to acknowledge that the limited sample size of this study precludes personalized risk stratification. Finally, while this study validates the independent prognostic significance of the residual CatLet score, it does not provide a direct comparison with the established residual SYNTAX score. Given that the SYNTAX score remains a widely used benchmark in clinical decision-making, future head-to-head studies are essential to determine whether the residual CatLet score offers superior discriminative power, calibration, or clinical net benefit for risk stratification in CCS patients after PCI. Such comparative studies will be crucial for defining the potential role of this new tool in clinical practice.

## 6 Conclusion

In this real-world PCI cohort, both the rCS and CRI effectively risk-stratified patients and predicted 6.2-year composite adverse cardiovascular events. However, their prognostic capacities were distinct.

## Data Availability

The original contributions presented in the study are included in the article/Supplementary Material, further inquiries can be directed to the corresponding authors.
